# Lurasidone Sub-Chronic Treatment During Adolescence Modulates Inflammatory and Inositol-Related Metabolic Pathways in the Prefrontal Cortex of Adult Male Rats Exposed to Prenatal Stress

**DOI:** 10.3390/biom16020327

**Published:** 2026-02-20

**Authors:** Monica Mazzelli, Samantha Saleri, Valentina Zonca, Moira Marizzoni, Marco Andrea Riva, Veronica Begni, Annamaria Cattaneo

**Affiliations:** 1 Laboratory of Biological Psychiatry, IRCCS Istituto Centro San Giovanni di Dio Fatebenefratelli, Via Pilastroni 4, 25125 Brescia, Italy; mmazzelli@fatebenefratelli.eu (M.M.); ssaleri@fatebenefratelli.eu (S.S.); mmarizzoni@fatebenefratelli.eu (M.M.); m.riva@unimi.it (M.A.R.); vbegni@fatebenefratelli.eu (V.B.); 2 Department of Pharmacological and Biomolecular Sciences, University of Milan, Via Balzaretti 9, 20133 Milan, Italy; valentina.zonca@unimi.it

**Keywords:** prenatal stress, prefrontal cortex, adolescence, lurasidone, inflammation, whole-genome expression analysis

## Abstract

Prenatal stress (PNS) predisposes individuals to mental disorders later in life. Adolescence is a period of heightened brain plasticity and vulnerability, when many mental disorders emerge, yet pharmacological strategies remain largely underexplored. In adult PNS rats, lurasidone (LUR) has been shown to reduce PNS-induced risk; however, its effects following adolescent administration remain unclear. To investigate the long-lasting effects of PNS and their modulation following sub-chronic LUR adolescent treatment, a whole-genome expression analysis of the prefrontal cortex (PFC) of adult male PNS rats was performed. Twelve PNS and eleven control rats were randomly assigned to receive vehicle or LUR from postnatal day (PND) 35 to 49 and sacrificed at PND 50. Partek Genomics Suite and Ingenuity Pathway Analysis were used for differential expression and pathway analyses. Within the PFC, PNS induced an upregulation of pathways involved in environmental information processing and in immune system-related pathways, which was reduced after LUR, as observed by IL-8 signaling (z-scores before: 1.34 and after LUR: −2.65). In parallel, LUR administration itself modulated Inositol-related metabolic pathways. Overall, these findings suggest that LUR adolescent treatment may counteract some PNS-induced alterations, supporting adolescence as a critical window for early preventive strategies with translational relevance for stress-related neuropsychiatric disorders.

## 1. Introduction

Early life exposure to stress, starting from the prenatal period, enhances vulnerability to psychiatric disorders later in life by influencing different biological processes, including neuroplasticity and inflammation [1]. Pregnant women suffering from depression showed increased blood levels of pro-inflammatory cytokines, which in turn were associated with altered cortisol secretion during pregnancy and similar biological patterns were also observed in the offspring [2,3,4]. Moreover, different stressors, such as socioeconomic disadvantages in pregnancy, combined with the presence of a pro-inflammatory status, have been associated with brain morphological abnormalities and behavioral alterations in offspring [5].

Rat offspring exposed to the prenatal stress (PNS) paradigm, that mimics the human adverse stressful conditions experienced during pregnancy, reported altered behaviors, including reduced locomotor activity and social preference, and enhanced depressive- and anxiety-like behaviors, starting from adolescence through adulthood [6,7,8,9]. PNS-exposed offspring also reported several brain alterations later in life. For example, increased levels of microglial activation markers and pro-inflammatory cytokines (such as Interleukin (IL)-1β, IL-6 and tumor necrosis factor-alpha, TNF-α) have been reported in the brains of adolescent and adult rats exposed to PNS [10,11,12,13]. Impairment in hippocampal neuroplasticity [14,15,16] along with cognitive impairment has been described in maternally stressed offspring, at adulthood, likely due to the inability of PNS-exposed animals to activate genes and pathways involved in cognitive processes [17]. One of the developmental periods during which PNS induced alterations was adolescence, a critical stage of emotional and cognitive development marked by profound changes in brain structure and function [18,19]. Accordingly, adolescence represents one of the most sensitive temporal windows for the development of mental disorders and is when many mental disorders first emerge [12,20].

Several attempts have been made to investigate the ability of pharmacological treatments to counteract or moderate the risk associated with the exposure to stress early in life on mental health [21,22]. Evidence from the literature suggests that long-lasting PNS exposure influenced the pattern of expression of several neurotransmitter receptors in rodents, particularly 5HT2A and D2 receptors, contributing to the development of stress-related psychiatric disorders [23,24]. The second-generation antipsychotic drug lurasidone (LUR) was selected for a sub-chronic treatment as its pharmacological profile targeted the receptors affected and dysregulated by PNS exposure [23,24]. Indeed, LUR is a multimodal antipsychotic drug able to act as an antagonist of dopamine D2 receptors and serotonin 5-HT2A and 5-HT7 receptors with high affinity, and as a partial agonist of the 5-HT1A receptors with moderate affinity [25]. Moreover, a chronic treatment with LUR normalized the behavioral impairment as well as the synaptic alterations observed in the prefrontal cortex (PFC) and hippocampus of adult rats exposed to PNS [24,26], due to its antidepressant [27,28] and pro-cognitive [25,29] properties. However, little is known about the effects of its administration during adolescence on long-term PNS-induced biological alterations. In the present study, we specifically selected adolescence, a highly dynamic period characterized by both vulnerability and resilience [30], as the experimental window to test whether early intervention can prevent long-term alterations induced by PNS and their subsequent psychological effects later in life.

This study aimed at dissecting the biological pathways affected in the PFC of adolescent animals exposed to PNS and at assessing whether a sub-chronic treatment with LUR during adolescence can prevent the biological alterations induced by PNS exposure. Consistent with the exploratory nature of the study, the analysis focused on pathway-level interpretation to generate hypotheses on the molecular effects of PNS and LUR.

## 2. Materials and Methods

### 2.1. Animals

Nulliparous adult female (body weight of 230–260 g) and male (400 g) Sprague-Dawley rats were purchased from a commercial breeder (Charles River, Calco, Italy). Upon their arrival, the rats were pair-housed with a same-sex conspecific animal with food and water available ad libitum. Animal handling and experimental procedures were conducted under the authorization from the Health Ministry (no. 295/2012-A of 20 December 2012), in full compliance with the Italian legislation on animal experimentation (Decreto Legislativo 116/92), in accordance with EU recommendation (EEC Council Directive 86/609 1987) and following the National Institute of Health Guide for the Care and Use of Laboratory Animals. All efforts were made to minimize animal suffering and to reduce the total number of animals used while preserving statistical validity.

#### 2.1.1. Prenatal Stress Paradigm

The PNS paradigm was performed following the protocol already published [12,31,32]. Briefly, as already mentioned Sprague-Dawley male and nulliparous female rats were housed in two animals per standard cages accordingly to sex and ten days after their arrival in the animal facility, male and female rats were mated for 24 h and individually housed immediately thereafter. Pregnant rats were randomly assigned to control (CTRL) or PNS condition. For the PNS-exposed group, pregnant dams were restrained into a transparent Plexiglas cylinder (20 cm length × 9 cm diameter × 9 cm height), under bright light (1500 lux), for 45 min, three times daily during the third and last week of gestation (gestational day, GD, 14) at varying times of the day to reduce habituation, whereas CTRL dams were left undisturbed in their home cages. On postnatal day (PND) 1, pups from CTRL and PNS dams were culled to 10 pups (5 males and 5 females). Pups were left undisturbed until PND 21 when they were weaned and housed in same-sex groups of 3 animals per standard cages with no environmental enrichment. No behavioral evaluation was performed on rats of the present preclinical experiment.

#### 2.1.2. Lurasidone Treatment

LUR (Sumitomo Pharma Co. Ltd., Tokyo, Japan) was prepared by suspending the compound at a concentration of 3 mg/mL in 1% Hydroxyethylcellulose. The drug or vehicle, VEH (1% Hydroxyethylcellulose) was administered daily by oral gavage. The selected dose and route of administration were based on previous studies reporting the antidepressant effects and pro-cognitive actions of LUR [24].

**Figure 1:**
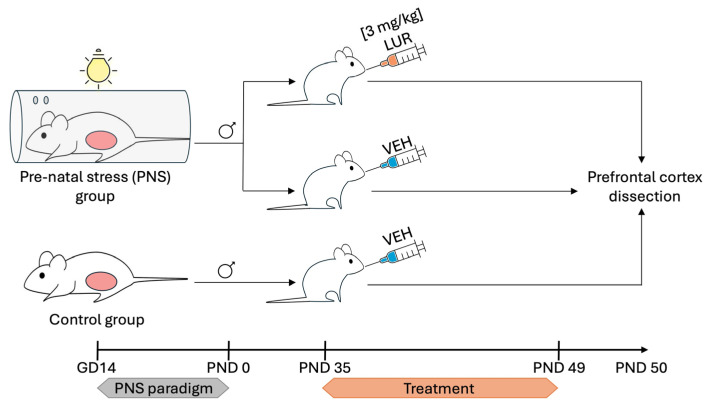
Experimental design. Pregnant rats were randomly assigned to CTRL or PNS conditions, with stress exposure paradigm started from gestational day (GD) 14. From PND 35, CTRL (n = 11) and PNS-exposed (n = 12) adolescent male offspring rats were randomized to receive daily VEH or LUR for two weeks (until PND 49). All the animals were sacrificed 24 h after the last drug treatment at PND 50 and PFC was dissected. For the analyses of the manuscript, the following experimental groups were included: CTRL-VEH, PNS-VEH, and PNS-LUR. Meanwhile the CTRL-LUR group was not considered. Specifically, the figure shows only the experimental groups that were included in the subsequent analyses.

Only male offspring was considered for the present experiment. During adolescence, starting at PND 35, CTRL (n = 11) and PNS-exposed (n = 12) male offspring rats were randomized to receive daily vehicle (VEH) or LUR for 2 weeks (until PND 49), resulting in four experimental groups: PNS-exposed male rats receiving LUR 3 mg/kg (n = 6) or VEH (n = 6) and CTRL male rats receiving LUR 3 mg/kg (n = 6) or VEH (n = 5). All animals were sacrificed by decapitation at PND 50 (24 h after the last drug treatment). The PFC was dissected according to the atlas of Paxinos and Watson, immediately frozen on dry ice and stored at −80 °C for the subsequent analyses. For the analysis included in this manuscript, a total of 17 animals were distributed across the following experimental groups: 5 CTRL rats receiving VEH (CTRL-VEH), 6 PNS-exposed rats receiving VEH (PNS-VEH), and 6 PNS-exposed rats treated with LUR (PNS-LUR), whereas the CTRL-LUR group was not included (Figure 1). The sizes of each experimental group matched those of comparable rodents’ transcriptomic studies.

### 2.2. RNA Isolation

Total RNA was extracted by using the PureZOL RNA isolation reagents according to the manufacturer’s protocols (Bio-Rad Laboratories, Hercules, CA, USA). RNA quantity was assessed using a Nanodrop spectrophotometer (NanoDrop Technologies, Wilmington, DE, USA), while RNA integrity has been evaluated with an Agilent Bioanalyzer 2100 (Agilent Technologies Inc., Santa Clara, CA, USA), and a RNA Integrity Number (RIN) > 8 was detected for all the samples.

### 2.3. Whole-Genome Expression Microarray Analyses

RNA samples isolated from the PFC were used for whole-genome transcriptomic analyses [12] with the Rat Gene 2.1st Array Strips, covering 27,147 coding transcripts to ensure methodological continuity with our previous work [12] where the microarray approach was performed. Briefly, 250 ng of total RNA was used to synthesize second-strand cDNA with the GeneChip^®^ WT PLUS Reagent Kit (Affymetrix, Santa Clara, CA, USA). The purified cDNA was, then, fragmented, and a total of 5.5 µg of fragmented cDNA was labeled and hybridized onto the Rat Gene 2.1st array strips. The reactions of hybridization, fluidics, and imaging were conducted on the Affymetrix Gene Atlas instrument according to the manufacturer’s protocol (Affymetrix, Santa Clara, CA, USA).

### 2.4. Statistical Analyses

Raw microarray data (.CEL files) were imported from the output files of GeneAtlas Affymetrix platform (Affymetrix, Santa Clara, CA, USA) and analyzed in terms of expression data analyses (quality controls and statistical analyses) with Partek Genomics Suite 6.6 software (Partek, St. Louis, MO, USA). The following comparisons were analyzed: PNS-exposed rats treated with VEH (PNS-VEH) versus CTRL rats treated with vehicle (CTRL-VEH) and PNS-exposed rats treated with LUR (PNS-LUR) versus PNS-VEH.

Principal-component analysis (PCA) was used to detect potential outliers and batch effects. Background correction was applied using the Robust Multi-strip Average (RMA) [33] method to remove noise from autofluorescence followed by quantile normalization [34] to normalize the distribution of probe intensities among different microarray chips. Finally, a summarization step was conducted using a linear median polish algorithm to integrate probe intensities and to compute the expression levels for each gene transcript. A maximum filter of *p*-value (*p*) < 0.05 (no FDR correction applied) and a minimum absolute fold change (FC) cut-off of 1.1 was used to select the significant differentially expressed (DE) transcripts. Statistical significance is therefore reported using *p*-values rather than FDR-corrected q-values. The microarray data (.CEL files) of 3 samples (1 from CTRL-VEH, 1 from PNS-VEH, and 1 from PNS-LUR) were detected as outliers based on quality control (PCA) and were not included in the statistical analysis.

Pathway analysis was performed using the “Core Analysis” function in the Ingenuity Pathway Analysis (IPA) tool, v.2024 (Ingenuity System Inc., Redwood City, CA, USA, http://www.ingenuity.com, accessed on 21 November 2025). A threshold of *p* < 0.05 and a minimum absolute z-score cut-off of 1.2 [35] was used to identify significant pathways. All the figures were generated in R using ggplot2 (v. 3.5.0) [36] and UpSetR (v. 1.4.0) [37].

## 3. Results

### 3.1. Transcriptomic Profiles and Biological Processes Modulated by PNS in the PFC of Adolescent Male Animals

**Figure 2:**
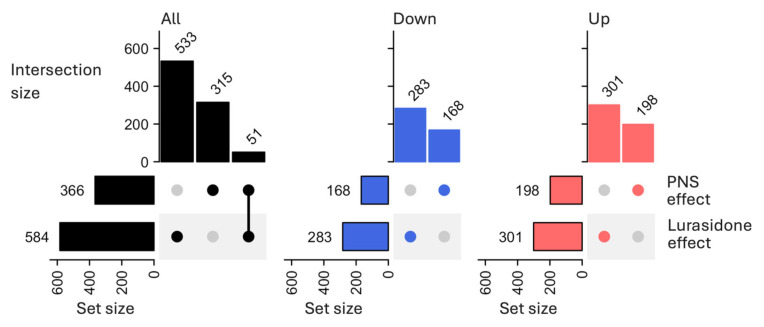
Transcriptional effects of PNS and lurasidone in PFC. UpSet plot summarizes the overlap of the differentially expressed (DE) transcripts modulated in PNS-VEH versus CTRL-VEH (PNS effect) with those modulated in PNS-LUR versus PNS-VEH (lurasidone effect). The DE transcripts were defined as *p* < 0.05 and |fold change (FC)| > 1.1. Color coding indicated the direction of the modulation: black for all DE transcripts, blue for downregulated (FC < −1.1) and red for upregulated (FC > 1.1) DE transcripts. The “Set size” bar graphs (bottom left) show the total number of DE transcripts identified in each comparison. Single circles in the matrix represent unique DE transcripts, while connected circles denote overlapping DE transcripts between comparisons. The top bar graph shows the corresponding number of DE transcripts for each unique or overlapping combination.

Our first aim was to identify alterations in the transcriptomic profile of the PFC of adolescent male rats exposed to PNS versus controls (PNS-VEH versus CTRL-VEH). We identified 366 differentially expressed (DE) transcripts, (|FC| > 1.1, *p* < 0.05), and out of them, 168 were downregulated and 198 were upregulated (Figure 2, Supplementary File S1).

**Figure 3:**
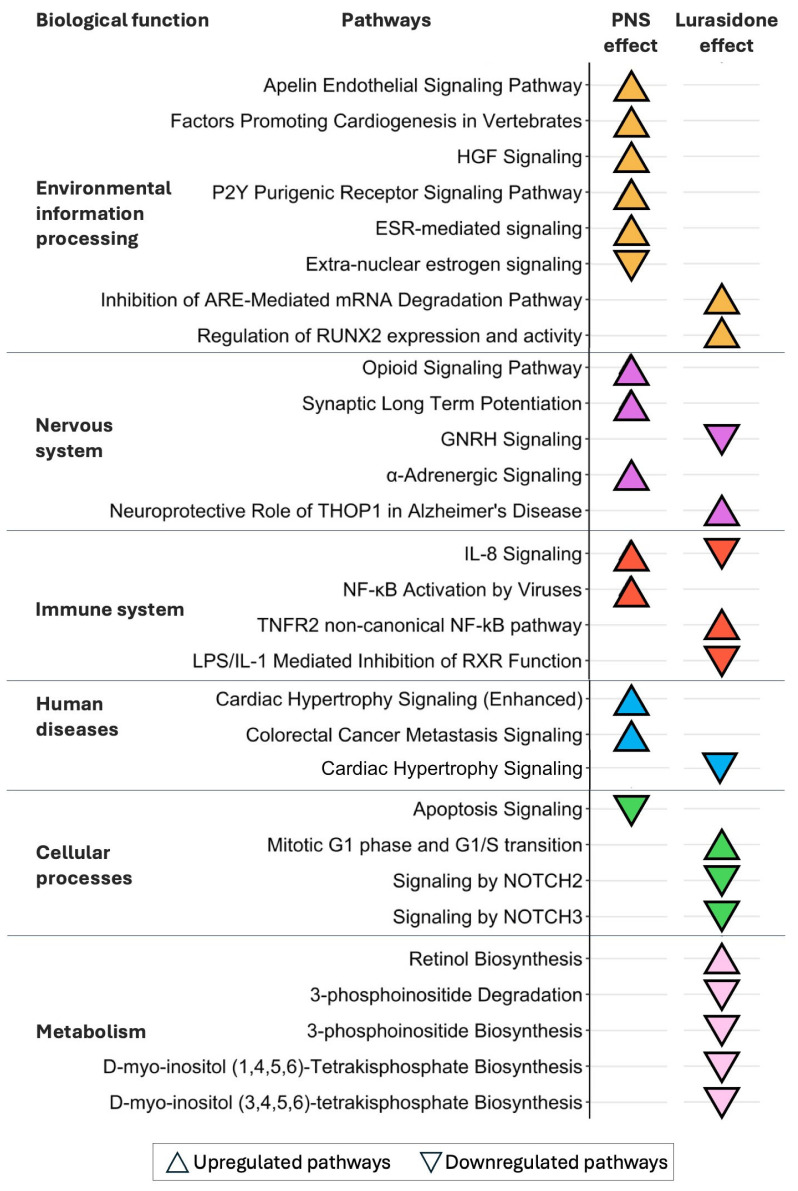
Biological analyses of PNS and lurasidone in PFC. The cuneiform plot is divided into six panels representing distinct pathway categories: environmental information processing, nervous system, immune system, human diseases, cellular processes and metabolism. Upregulated pathways (z-score > 1.2, *p* < 0.05) are shown as upward-facing triangles, while downregulated pathways (z-score < 1.2, *p* < 0.05) are shown as downward-facing triangles. The absence of a triangle indicates the absence of significant modulation.

Pathway analyses revealed that PNS exposure significantly modulated 14 pathways, 43% of which were associated with environmental information processing, 22% with nervous system, 14% with immune system, 14% with human diseases and 7% with cellular processes, according to KEGG definition (Figure 3, “PNS effect” column, Supplementary File S3).

Notably, 86% of the pathways modulated by PNS were upregulated, and among the most significant ones we reported an upregulation of immune pathways associated with the IL-8 signaling and the nuclear factor kappa-light-chain-enhancer of activated B cell (NF-kB) activation by viruses (z-score = 1.34 for both) processes. By contrast, only 14% of pathways appeared to be downregulated, and they included apoptosis in cellular processes (z-score = −2.00).

### 3.2. Sub-Chronic Lurasidone Treatment During Adolescence Normalized the Long-Lasting Effects of PNS Exposure

To evaluate the effect of LUR on PNS-induced molecular changes in the PFC, we compared the transcriptomic profile of PNS-exposed rats treated with LUR with those of PNS-exposed rats receiving the vehicle (PNS-LUR versus PNS-VEH). A total of 584 significant DE transcripts (283 downregulated and 301 upregulated) were identified (Figure 2, Supplementary File S2) and pathway analysis revealed that, among the 16 significant biological pathways identified, 31% were associated with metabolism, 19% with immune system, 19% with cellular processes, 13% with nervous system, 13% with environmental information processing, and 5% with human diseases (Figure 3, “Lurasidone effect” column, Supplementary File S4). Out of these, 40% were upregulated and included immune system-related processes, such as the tumor necrosis factor receptor 2 (TNFR2) non-canonical NF-kB pathway (z-score = 2.24), while the 60% were downregulated. Among those downregulated, we found the IL-8 signaling pathway (z-score = −2.65) and metabolic processes related to inositol metabolism, such as the 3-phosphoinositide degradation (z-score = −1.27) and biosynthesis (z-score = −1.51) and the D-myo-inositol (1,4,5,6) or (3,4,5,6)-tetrakisphosphate biosynthesis (z-score = −1.27 for both).

Finally, we evaluated the pathways modulated by PNS (Figure 3, “PNS effect”) and by LUR in PNS-exposed rats (Figure 3, “Lurasidone effect”) to disentangle the effects of LUR treatment from those induced by PNS exposure. Specifically, LUR effects extended to the pathway not affected by PNS, including the downregulation of inositol metabolism and of the neurogenic locus notch homolog protein, NOTCH 2 and 3, signaling (z-score < −2.00, for both). Additionally, LUR treatment completely reversed the effect of PNS on the IL-8 signaling pathway (z-score = 1.34 after PNS exposure, z-score = −2.65 after LUR treatment). To further address this opposite modulation, we examined transcript-level modulation of DE transcripts belonging to IL-8 signaling pathway in both conditions. Interestingly, in PNS rats we found an upregulation of the following genes: Ccnd1 (FC = 1.14), Gna11 (FC = 1.15), Nfkb1 (FC = 1.14), Prkce (FC = 1.12), and Prkcg (FC = 1.28). Conversely, Bax (FC = −1.12), Eras (FC = −1.10), Gng12 (FC = −1.13), and Gng5 (FC = −1.19) resulted in downregulation. Collectively, the expression of these genes results in a significant activation of the IL-8 signaling pathway. Following LUR treatment, we found all the genes modulated to be downregulated: Flt1 (FC = −1.13), Fnbp1 (FC = −1.11), Fos (FC = −1.52), Gna11 (FC = −1.11), Gng13 (FC = −1.24), Itgb2 (FC = −1.17), Jun (FC = −1.29), Kras (FC = −1.23), Rab11fip2 (FC = −1.10), and Rhot1 (FC = −1.32). This results in a downregulation of this pathway. Notably, Gna11, a gene belonging to the family of G-proteins involved as modulators in different intracellular signaling systems, was the only DE transcript modulated in both conditions, and exhibited opposing regulation.

## 4. Discussion

In this study, we identified the molecular transcriptomic profile associated with prenatal stress exposure in the PFC of male rats during adolescence, a critical period for the development of this brain region and a vulnerable temporal window for the onset of mental disorders [12,20]. We have also evaluated how these molecular transcriptomic signatures could be modulated by LUR treatment, when administered during adolescence.

### 4.1. Long-Term Alterations of PNS

This work confirms and extends our earlier findings that PNS exposure induced long-term alterations in the PFC mainly through activation of the inflammatory/immune system response [12], as indicated by the upregulation of the IL-8 signaling and the NF-kb activation by viruses pathways, as well as in molecular processes with functional relevance in microglial reactivity, such as the Apelin endothelial signaling [38], the P2Y purigenic receptor signaling [39] and the Hepatocyte Growth Factor (HGF) signaling [40] pathways. PFC is strongly interconnected with the hippocampus and the amygdala, and this network is essential to regulate mood, emotions, and stress responsiveness [41,42]. During adolescence, PFC undergoes a massive synaptic pruning by microglia to improve the efficiency of the cortical brain network [43]. Early life adversities cause a disruption in the developing microglia, leading to a primed state that responds excessively to further immune challenges [44]. In this context, our findings support the idea that dysregulation of the PFC–amygdala–hippocampus transmission may be a long-term signature of PNS exposure that ultimately leads to increased risk for neuropsychiatric disorders in the offspring [20] via neuroinflammation.

Our pathways analysis further revealed the upregulation of signatures associated with neurotransmission, such as the alpha-Adrenergic and the Opioid signaling pathways. The former seems to contribute to stress-induced impairments in PFC cognitive and emotional functions [45,46]. Similarly, the latter plays an essential role in stress-related emotional and physiological responses, and its activation has been associated with the impairment of memory retrieval induced by stressful events [41]. Moreover, the binding of opioids to their receptors has been recently shown to activate the Toll-like receptor (TLR) 4 and to enhance the release of pro-inflammatory cytokines in the brain [42], further emphasizing the involvement of the immune system.

### 4.2. Beneficial Effects of LUR Treatment

Our results demonstrated the efficacy of a sub-chronic LUR treatment during adolescence in counteracting the PFC alterations induced by PNS exposure. We found that all the pathways modulated by PNS were no longer up/downregulated after LUR treatment. Interestingly, our data have identified several novel LUR-responsive transcripts, including those involved in the IL-8 signaling pathway which was modulated in the opposite direction by LUR as compared with PNS. The analysis of DE transcripts belonging to the IL-8 signaling revealed an overall upregulation of the transcripts, consistent with the activation of the pathway following PNS exposure. Conversely, all the transcripts modulated following LUR treatment were downregulated, suggesting instead an inhibition of IL-8 signaling. Among the upregulated genes belonging to the IL-8 signaling following PNS, Nfkb1 was identified. Interestingly, Nfkb1 encodes a transcription factor known to be strongly associated with inflammation through the regulation of cytokines and chemokines [47]. On the contrary, among the IL-8 signaling-related genes downregulated following LUR sub-chronic treatment, Flt1 was of particular interest. Flt1 is involved in the regulation of neurogenesis, cognitive impairment, and angiogenesis. Interestingly, in their study Zhang and colleagues showed that the inhibition of Flt1 in the offspring of female rats exposed to PNS was associated with the improvement of neurodevelopmental abnormalities [48]. This evidence is in line with our results suggesting that the reduction of Flt1 may, at least in part, drive the therapeutic effects of LUR. Although the opposite modulation of the IL-8 signaling as a consequence of PNS exposure and LUR sub-chronic treatment did not reflect a complete gene-by-gene counter modulation (as the only gene shared between the two conditions was Gna11), the opposite modulation (due to PNS or LUR) of the IL-8 signaling pathway supported the hypothesis that lurasidone was able to counteract the inflammatory effects exerted by PNS exposure on the pathway. These novel findings on the potential anti-inflammatory effect of LUR are in line with previous studies showing that various psychotropic drugs can influence central inflammation [49,50,51]. Antidepressant drugs have been reported to reduce neuroinflammation through the NF-kB pathway, lowering cytokine levels both in rodents and humans [52,53]. For instance, the selective serotonin reuptake inhibitor fluoxetine given during the perinatal period exerted a protective effect on anxiety and depressive-like behaviors as well as on hypothalamic–pituitary–adrenal axis functionality and reverted the PNS-induced negative effects on neuroplasticity and inflammation in PNS-exposed offspring [54]. Besides central effects, clinical studies also demonstrated decreased levels of pro-inflammatory mediators like IL-6 and TNF-α in blood samples from patients responding to antidepressant treatment [55,56,57,58]. The modulatory effect of LUR on the immune system is further supported by the downregulation of the cellular process-related pathways linked to the NOTCH2 and NOTCH3 signaling. The canonical Notch signaling pathway was associated with synaptic plasticity and inflammation in the central nervous system. NOTCH2 in the PFC promoted the activation of the NF-kB pathway and contributed to the development of depressive-like behaviors in chronic-social-defeat-stress mice and Wistar Kyoto rats [59]. Similarly, the activation of the NF-kB pathway by NOTCH3 promoted the expression of pro-inflammatory genes in TLR-4 activated macrophages [60]. These data suggest that the anti-inflammatory properties of LUR may account for its therapeutic properties in the context of early-life stress exposure.

Finally, as for other antipsychotics such as lithium [61] and olanzapine [62], we found a modulatory effect of LUR on inositol-related pathways. Inositol is a sugar-like compound [52,63] involved in a plethora of biochemical functions such as metabolic, endocrine modulation and signal transduction [64,65]. LUR was found to have high binding affinity for 5-HT7 and 5-HT2A receptors [66], which are coupled to Gq proteins and influence the GPCR-linked phospholipase C (PLC) activity [67] PLC is responsible for the generation inositol-1,4,5-trisphosphate (IP_3_) [68], the precursor of higher phosphorylated inositol polyphosphates, that regulate neuronal differentiation and cell signaling. IP_3_ also modulates the PI3K/Akt cascade, a key regulator of neuroinflammatory responses [69]. Through these pathways, LUR could attenuate neuroinflammation via altered inositol metabolism; however, specific mechanisms linking LUR, inositol metabolism and inflammation need to be further investigated.

Overall, our findings highlight biological mechanisms that may be highly relevant in clinical contest, namely inflammation. Indeed, elevated cytokines and immune-system signaling are consistently linked to mood disturbances [70,71] in adolescents exposed to early-life stress, and interventions that modulate inflammation show promising results for alleviating depressive symptoms [72,73,74]. Accordingly, we found that LUR could modulate immune-related pathways, suggesting a potential mechanism that aligns with current translational efforts, although further validation in clinical cohorts is required.

In our study some limitations should be mentioned. First, the experiments were conducted exclusively in males, although we are aware that stress can induce sex-specific alterations. Particularly during adolescence, when the influence of sex hormones is most pronounced, sex-dependent effects emerge across depression-like behavior, HPA-axis activity, and pro-inflammatory cytokines [10,75,76,77]. Collectively, these data indicate that male-only findings may underestimate stress-induced phenotypes in females and as a future perspective, we aim to extend these investigations to female cohorts to directly assess potential sex-specific effects. Second, behavioral data are missing; therefore, we cannot correlate changes in the inflammatory signatures with long-term behavioral consequences associated with PNS or with a beneficial effect of LUR. Lastly, transcriptomic analyses have been performed by using the microarray technology rather than the more advanced RNA sequencing approach to ensure methodological continuity with our previous work [12] where the microarray approach was performed. Lastly, another limitation is the lack of a comprehensive transcriptomic analysis of the CTRL-LUR vs. CTRL-VEH comparison.

## 5. Conclusions

In conclusion, this study confirms that prenatal stress induces long-lasting transcriptional changes in the prefrontal cortex, impacting the immune system and metabolism. Additionally, our findings highlight novel anti-inflammatory properties of lurasidone, which may underlie its effectiveness in mitigating or reversing the enduring effects of early-life stress. Further research is needed to validate these anti-inflammatory effects and explore the broader therapeutic potential of this antipsychotic. Future studies could further investigate the molecular mechanisms underlying lurasidone’s anti-inflammatory effects, evaluate its long-term efficacy across different models of early-life stress, and explore potential behavioral and cognitive outcomes to fully assess its therapeutic potential also in the clinical setting.

## Data Availability

The original contributions presented in this study are included in the article/Supplementary Material. Further inquiries can be directed to the corresponding author(s).

## Supplementary Materials

The following are available online at https://www.mdpi.com/article/10.3390/biom16020327/s1: Supplementary File S1: List of transcripts modulated by PNS; Supplementary File S2: List of transcripts modulated by lurasidone; Supplementary File S3: List of pathways and biological functions affected by PNS; Supplementary File S4: List of pathways and biological functions affected by lurasidone.

## Abbreviations

The following abbreviations are used in this manuscript:

Bax	BCL2-Associated X, Apoptosis Regulator
BDNF	Brain-Derived Neurotrophic Factor
Ccnd1	Cyclin D1
CTRL	Control
DE	Differentially expressed
Eras	ES Cell-Expressed Ras
FC	Fold change
Flt1	Fms-Related Receptor Tyrosine Kinase 1 (VEGFR-1)
Fnbp1	Formin Binding Protein 1
Fos	Fos Proto-Oncogene, AP-1 Transcription Factor Subunit
Gna11	G Protein Subunit Alpha 11
Gng5	G Protein Subunit Gamma 5
Gng12	G Protein Subunit Gamma 12
Gng13	G Protein Subunit Gamma 13
HGF	Hepatocyte Growth Factor
IL	Interleukin
IP_3_	Inositol-1,4,5-trisphosphate
IPA	Ingenuity Pathway Analysis
Itgb2	Integrin Subunit Beta 2
Jun	Jun Proto-Oncogene, AP-1 Transcription Factor Subunit
Kras	KRAS Proto-Oncogene, GTPase
LUR	Lurasidone
NF-κB	Kappa-light-chain-enhancer of activated B cells
Nfkb1	Nuclear Factor Kappa B Subunit 1
NOTCH	Neurogenic locus notch homolog protein
PCA	Principal-component analysis
PND	Postnatal day
PFC	Prefrontal cortex
PLC	Phospholipase C
PNS	Prenatal stress
Prkce	Protein Kinase C Epsilon
Prkcg	Protein Kinase C Gamma
RIN	RNA Integrity Number
Rab11fip2	RAB11 Family Interacting Protein 2
Rhot1	Ras Homolog Family Member T1 (Miro1)
RMA	Robust Multi-strip Average
TLR	Toll-like receptor
TNF-α	Tumor necrosis factor-alpha
TNFR2	Tumor necrosis factor receptor 2
VEH	Vehicle
